# Comparative analysis of productive performance and fattening efficiency of commercial pigs in China for two consecutive years

**DOI:** 10.1038/s41598-023-35430-y

**Published:** 2023-05-19

**Authors:** Ran Guan, Junqiang Wu, Yunzhou Wang, Qian Cai, Xiaowen Li

**Affiliations:** 1 Shandong New Hope Liuhe Agriculture and Animal Husbandry Technology Co., Ltd (NHLH Academy of Swine Research), No. 6596 Dongfanghong East Road Yuanqiao Town, Dezhou, 253000 Shandong People’s Republic of China; 2 Shandong New Hope Liuhe Group Co., Ltd., No. 592-26, Jiushui East Road, Laoshan District, Qingdao, Shandong People’s Republic of China; 3 Xiajin New Hope Liuhe Agriculture and Animal Husbandry Co., Ltd, Guozhai Forest Farm, Suliuzhuang Town, Xiajin County, Dezhou, Shandong People’s Republic of China; 4 Jiaozhou Customs, No. 1 Changjiang Road, Qingdao, 266300 Shandong People’s Republic of China; 5 Swine Health Data and Intelligent Monitoring Project Laboratory, Dezhou University, No. 566 University Road West, Decheng District, Dezhou, 253023 Shandong People’s Republic of China; 6 Healthy Breeding of Swine and Poultry and Disease Diagnostic Technique Engineering Laboratory in Shandong Province, No. 592-26 Jiushui East Road Laoshan District, Qingdao, 266100 Shandong People’s Republic of China

**Keywords:** Risk factors, Biomarkers

## Abstract

The purpose of this study: (1) propose an evaluation indicator of the fattening efficiency of commercial pigs (Yorkshire × Landrace × Duroc)—fattening efficiency index (FEI). (2) Analyze the correlation to find the main productive factors affecting the FEI. (3) Compare and analyze the yearly/monthly/different piglets’ sources of productive performance in 2020 and 2021. The data included 2592 commercial pig batches in 2020 and 3266 in 2021, with a total of 6,134,234 commercial pigs. Descriptive statistics and difference analysis were carried out on 16 productive factors of a whole year and single/multiple sources for two consecutive years. The same period difference between the monthly data and the annual average were also analyzed. The top six productive factors correlated with FEI were average daily gain (ADG) (0.8080), feed conversion rate (FCR) (− 0.7203), survival rate (SR) (0.6968), number of deaths (− 0.4103), feeding days (− 0.3748) and body weight (BW) of marketing pigs (0.3369). The overall productive performance in 2021 was lower than that in 2020, which was reflected in more piglet sources and a lower BW of piglets, more deaths, a lower SR, longer feeding days, a lower ADG, a higher FCR and a lower FEI. The productive performance of a single source was better than that of multiple ones. The contrastive results of monthly data in 2020 and 2021 showed significant differences in most factors except for the number of marketing pigs, the number of piglets and feed consumption. The monthly trend of 15 factors for two consecutive years revealed similar trends only in the month of piglets purchasing, number of piglets sources, number of deaths and ADG. Compared with the annual average, the ADG significantly increased in May. The FEI of multiple sources was markedly lower than that of a single source. FEI may be suitable for evaluating the fattening efficiency of commercial pigs. The annual and monthly productive performance and fattening efficiency in 2021 were significantly lower than those in 2020. Single source was represented better productive performance and fattening efficiency than multiple ones.

## Introduction

Commercial pigs are generally from three-way crossbred (such as Yorkshire × Landrace × Duroc), referring to live pigs produced and sold to provide pork. Given the economic and social benefits, pork is considered a global strategic product, which is the second most widely produced meat product in the world^1^. Pig herds with higher productive efficiency not only have lower feed conversion rate (FCR), but also produce less global warming, acidification and eutrophication^2^. In practice, piglets weaned per sow per year (PSY) is generally used as the evaluation index of sow reproductive efficiency^3^. However, no comprehensive indicator has been found to evaluate the fattening efficiency of commercial pigs.

In August 2018, African Swine Fever (ASF) broke out in China, which was a highly contagious and lethal viral disease^4^. Within 8 months, the ASF spread to all Chinese mainland, and 1 year later, the stock of commercial pigs and sows dropped by about 40%^5^. To stabilize pig production and ensure market supply, in March 2019, the Ministry of Agriculture and Rural Affairs of China announced and implemented a series of policies, focusing on supporting breeding and large-scale pig farms to resume production as soon as possible^6^. As a result, China has sold 671.28 million pigs in 2021 (an increase of 27.4% more than in 2020), with the pork production being 529.6 million tons (an increase of 28.8% more than in 2020)^7^. However, the fattening efficiency of commercial pig batches under large-scale pig farms has not been evaluated. Therefore, this study proposed an evaluation index of the productive efficiency of commercial pigs—the fattening efficiency index (FEI). The productive data of commercial pigs from 2592 batches in 2020 and 3266 batches in 2021 were analyzed and further compared to the annual and monthly data. The possible reasons for the lower productive efficiency in 2021 were proposed to provide references for pig farm managers to formulate and improve the productive efficiency of commercial pigs.

## Methods

The study did not require approval from the Ethics Committee on Animal Use because no animal was handled.

### Farm description

All pig farms studied (n = 2149 in 2021; n = 2176 in 2020) were from a domestic large-scale pig company in China. The farms fulfilled the following inclusion criteria. (1) There were no other pig farms within 500 m around. (2) Zones of bio-security management: the periphery, productive area, living area and fecal sewage treatment area were separated by walls of more than 1.8 m. (3) The living area had offices, dormitories, warehouses, canteens and other rooms. (4) There were independent sewage treatment areas and facilities. (5) It had a sanitary corridor with a bathroom for staff and a soaking disinfection room for goods. (6) The design scale of the hoggery was more than 700 heads. (7) There were feeding towers and automatic feeding lines to meet the maximum feed intake of pigs in 7 days. (8) Pigs were fed with the corresponding formula of standardized feed (10 kinds of feeds in the stages of nursery, growth, and fattening) provided by the company's internal feed factory. (9) There were water sources and water storage equipment that met the breeding water standards. (10) There were fences (about 0.9 m high) around the pens where the fattening pigs were located and concrete grids with sufficient opening width in the pig house. (11) The pig fattening facilities were equipped with an automatic environmental control system and mechanical ventilation system (climate controller for controlling fans of different sizes). (12) The pig farm had experienced veterinarians, breeders and other staff. (13) The pig farms used the internal data management system of the company.

**Figure 1:**
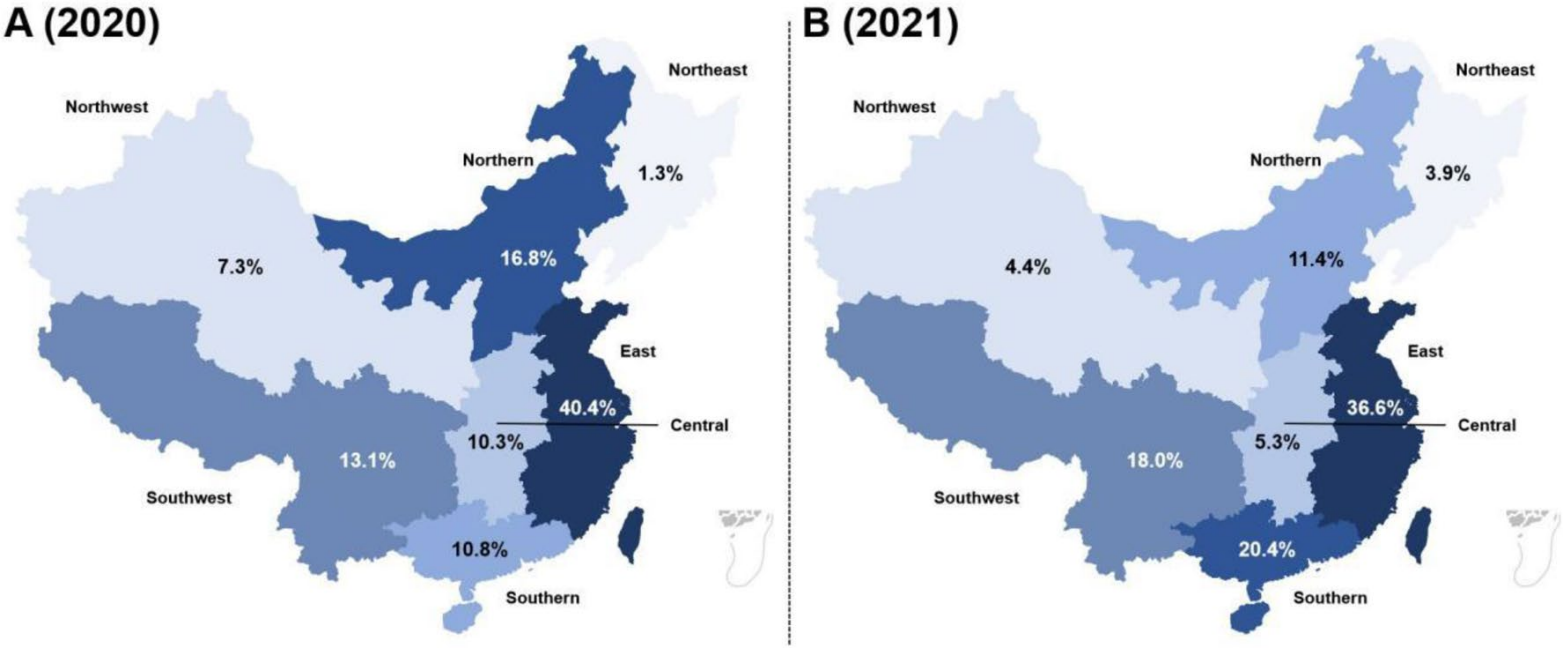
The distribution proportion of the batches in 2020 (**A**) and 2021 (**B**) in China’s seven major geographical regions. The darker the color, the higher the proportion.

The farms in 2020 were from 20 provinces (or municipality directly under the Central Government), while the farms in 2021 were from 21 provinces (or municipality directly under the Central Government). The distribution proportion of the batches in China's seven major geographical regions is shown in Fig. 1.

### Data collection and manipulation

The productive data were uploaded to the internal data management system by each pig farm. All data belonged to the company. The researchers were authorized by the company's productive management department and digital technology department to obtain the productive data for this study. Batch data with the following conditions was considered illogical and deleted. Body weight (BW) of piglets was less than 4 kg or more than 15 kg. (2) BW of marketing pigs was less than 65 kg. (3) Survival rate (SR) was less than 65%. (4) Feeding days were less than 120 days. (5) Number of piglets was less than 100. (6) Number of marketing pigs was less than 100. (7) Age of piglets was less than 18 or more than 70 days. (8) FCR was not 2–5. (9) ASF occurred. (10) Incomplete data records. Finally, 2,794,806 commercial pigs from 2592 batches from 90 companies in 2020 and 3,339,428 commercial pigs from 3266 batches from 122 companies in 2021 were selected.

The above data were processed and analyzed. Firstly, descriptive statistics of the productive performance of 2592 batches in 2020 and 3266 batches in 2021 were handled, and the data differences between 2020 and 2021 were analyzed. Secondly, the monthly differences between the data of these 2 years were compared, and the differences between the monthly data of 15 factors and the annual average value were analyzed. Then, the correlation coefficient was used to analyze the correlation among 17 productive factors, so as to find the factors related to the FEI. Finally, descriptive statistics and difference analyses were carried out for single and multiple sources of piglets in 2020 and 2021.

### Definitions

FEI was a comprehensive evaluation indicator of the three most important productive and health management indicators [FCR, SR, and average daily gain (ADG)] for commercial pigs under a basically reasonable SR during the feeding BW of 5–150 kg. It referred to the BW of commercial pigs converted from every kilogram of feed per day.

$${\text{FEI }}\left( {\text{g}} \right) \, = {\text{ SR }} \times {\text{ ADG }}\left( {{\text{kg}}} \right) \, \times { 1}000 \, /{\text{ FCR}}$$

According to the productive target of the company's commercial pigs, SR was 95%, ADG was 700 g, and FCR was 2.60. The marketing target value of FEI was 255.77 g. The reference growth performance and corresponding FEI of commercial pigs at corresponding feeding stages are presented in Table 1.

**Table 1:** Reference growth performance and FEI of commercial pigs at corresponding feeding stages.

Feeding stages	BW (kg)	Age (days)	ADG (kg)	ADFI (kg)	FCR	SR (%)	FEI (g)
Nursery	6–7.9	24–37	0.14	0.22	1.54	98.00	90.79
	8–13.9	38–54	0.35	0.48	1.37	97.00	249.77
	14–23.9	55–75	0.48	0.88	1.85	96.10	247.31
Growth	24–33.4	76–89	0.68	1.32	1.95	95.00	330.96
	33.5–43.4	90–103	0.71	1.62	2.26	94.00	296.88
Fattening	43.5–54.4	104–118	0.73	1.78	2.43	93.40	281.89
	54.5–66.4	119–133	0.80	2.12	2.66	92.60	278.95
	66.5–83.4	134–152	0.89	2.49	2.78	92.40	297.21
	83.5–106.9	153–177	0.94	2.96	3.15	92.20	275.17
	107–130	178–204	0.85	3.20	3.75	92.00	208.74
Average			0.70	1.71	2.60	95.00	255.77

*BW* body weight, *ADG* average daily gain, *ADFI* average daily feed intake, *FCR* feed conversion rate, *SR* survival rate, *FEI* fattening efficiency index.

The genotype of commercial pigs in this study was three-way crossbred (Yorkshire × Landrace × Duroc). The “marketing pig” referred to the fattening pigs weighing 110–130 kg for slaughter. The meaning of “purchasing piglet” was the purchase of weaned piglets aged around 21 days from the sow farms. The “SR” represented the survival rate of a batch of wean-to-finish pigs. The “single source” meant the piglets in the batch were all from one sow farm, while the “multiple sources” meant the piglets in the batch were merged from more than one sow farm.

### Statistics analysis

Descriptive statistics and correlation analysis were conducted using WPS Office Excel for Windows version 11.1.0.11830 (Kingsoft Office Corporation, Beijing, China). All difference analyses were conducted with Graphpad Prism 8.4 (Graphpad Software, Inc., San Diego, CA, USA). Unpaired *t* test was used for data difference analysis between 2020 and 2021 and single source vs. multiple sources. Dunnett's multiple comparisons test was used to analyze the difference between monthly data and the annual average.

### Ethics approval and consent to participate

All data in this study came from the internal data management system, and the author had access rights. The study only involved statistical analysis of the data, without field investigation of pig farms.

## Results

According to the combined calculation of 2592 batches in 2020 and 3266 batches in 2021, the top six productive factors related to FEI were ADG (0.8080), FCR (− 0.7203), SR (0.6968), number of deaths (− 0.4103), feeding days (− 0.3748) and BW of marketing pigs (0.3369) (Table 2). High correlation (> 0.8000) included number of marketing pigs versus number of piglets (0.9890), number of marketing pigs versus feed consumption (0.9562), feed consumption versus number of piglets (0.9499), and month of piglets purchasing versus month of marketing (0.8661). Moderate correlation (0.5000–0.8000) included number of deaths versus number of piglets (0.7653), number of deaths versus number of marketing pigs (0.6615), number of deaths versus feed consumption (0.6539), BW of marketing pigs versus ADG (0.6076), number of deaths versus SR (− 0.5867), feeding days versus BW of marketing pigs (0.5705) and age of piglet versus BW of piglets (0.5642). The weak correlation (0.3000–0.5000) included piglets sources versus BW of piglets (0.4872), piglets sources versus number of death (0.4647), SR versus ADG (0.4562), piglets sources versus number of piglets (0.4362), piglets sources versus number of marketing pigs (0.4014), piglets sources versus feed consumption (0.4761), FCR versus ADG (− 0.3691), ADG versus number of deaths (− 0.3418), SR versus BW of deaths (0.3268) and feeding days versus FCR (0.3176).

**Table 2:**
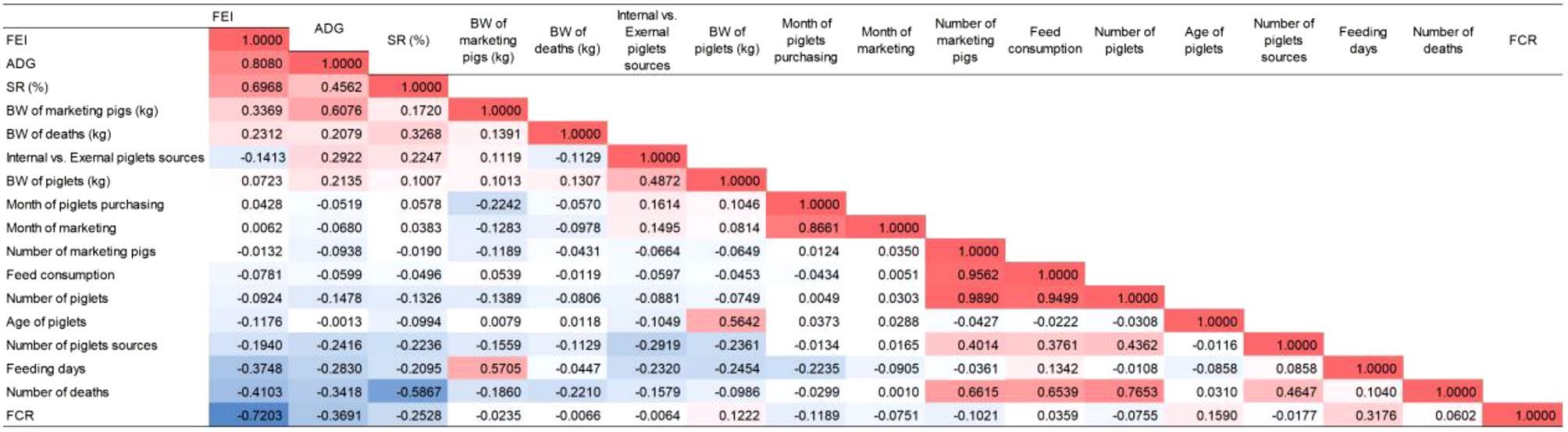
Correlation coefficient matrix of 17 productive factors in 5858 commercial pig batches.

Red represents positive correlation and blue represents negative correlation. The darker the color, the higher the correlation coefficient.

*FEI* fattening efficiency index, *ADG* average daily gain, *SR* survival rate, *BW* body weight, *FCR* feed conversion rate.

Table 3 showed that the overall productive performance in 2021 was significantly different from that in 2020, except for the number of piglets, feed consumption and BW of marketing pigs. Compared with 2020, the month of piglets purchasing in 2021 was 3.3 months earlier, piglet sources was 0.4 more, the BW of piglets was 0.8 kg lighter, the age of piglets was 5.6 days older, the number of deaths was 60.6 heads more, the BW of deaths was 3.7 kg lighter, and the month of marketing was 1.8 months earlier. The SR decreased by 4.8%, the number of marketing pigs was 55.7 less, the feeding days were 13.9 days more, the ADG was 0.06 kg less, the FCR was 0.14 higher, and the FEI decreased by 66.8 g.

**Table 3:** Productive performance of 2592 batches of commercial pigs in 2020 and 3266 batches in 2021.

	Month of piglets purchasing^a^		Number of piglets sources		Number of piglets		BW of piglets	
	2020	2021	2020	2021	2020	2021	2020	2021
Purchased piglets								
Average	0.7	− 2.6	1.3	1.7	1200.0	1204.9	8.3	7.5
SD	6.5	6.5	0.8	1.3	960.4	984.9	2.3	1.8
Median	3.0	1.0	1.0	1.0	950.0	941.5	7.8	7.0
Min	− 5.0	− 5.0	1.0	1.0	120.0	100.0	4.0	4.1
Max	8.0	8.0	10.0	14.0	9,448.0	10,960.0	15.0	15.0
*P*value	< 0.0001****		< 0.0001****		0.8510		< 0.0001****	

	Piglets age		Feed consumption		Number of deaths		BW of deaths^b^	
	2020	2021	2020	2021	2020	2021	2020	2021
Feeding								
Average	27.8	33.4	337,241.3	338,647.7	121.8	182.4	37.8	34.1
SD	9.0	9.4	259,824.5	266,995.3	159.1	211.4	16.6	17.3
Median	24.0	32.0	260,000.0	265,475.0	68.0	117.0	35.4	31.3
Min	18.0	18.0	28,420.0	25,660.0	0.0	0.0	5.4	2.1
Max	69.0	70.0	2,898,320.0	3,617,130.0	2,910.0	2,792.0	111.8	179.7
*P *value	< 0.0001****		0.8394		< 0.0001****		< 0.0001****	

	Month of marketing		SR		Number of marketing pigs		BW of marketing pigs	
	2020	2021	2020	2021	2020	2021	2020	2021
Marketing								
Average	8.5	6.7	90.6%	85.8%	1,078.2	1,022.5	120.1	119.9
SD	3.3	3.4	6.8%	8.5%	850.8	823.2	15.0	16.4
Median	9.5	7.0	92.5%	87.4%	838.5	786.0	121.0	120.5
Min	1.0	1.0	65.3%	65.0%	111.0	100.0	65.3	65.8
Max	12.0	12.0	100.0%	100.0%	8553.0	9589.0	179.6	171.2
*P *value	< 0.0001****		< 0.0001****		0.0112*		0.5864	

	Feeding days		ADG		FCR		FEI	
	2020	2021	2020	2021	2020	2021	2020	2021
Growth performance								
Average	164.5	178.4	0.68	0.62	2.78	2.92	225.8	189.0
SD	17.3	21.8	0.08	0.07	0.36	0.44	48.6	46.7
Median	164.0	178.0	0.68	0.63	2.72	2.85	230.5	190.0
Min	120.0	120.0	0.31	0.32	2.00	2.00	47.2	46.3
Max	263.0	271.0	0.90	0.89	4.99	5.00	374.2	360.8
*P *value	< 0.0001****		< 0.0001****		< 0.0001****		< 0.0001****	

*BW* body weight, *SR* survival rate, *ADG* average daily gain, *FCR* feed conversion rate, *FEI* fattening efficiency index.

^a^Month of piglets purchasing from last year was recorded as a negative number, the earlier the smaller.

^b^When number of deaths was zero, BW of deaths was recorded as blank.

*indicated *P* < 0.05; **indicated *P* < 0.01; ***indicated *P* < 0.001; ****indicated *P* < 0.0001.

**Figure 2:**
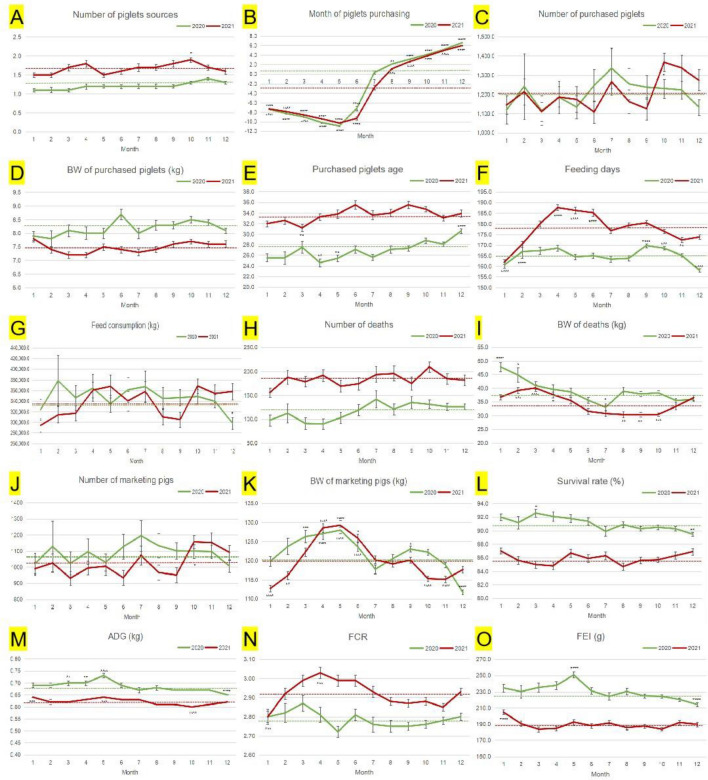
Monthly analysis of productive performance of 2592 batches of commercial pigs in 2020 and 3266 batches in 2021. The green dotted line indicated the average value in 2020 (n = 2592). The red dotted line indicated the average value in 2021 (n = 3266). The asterisk of 2020 was marked above the error line, and the asterisk of 2021 was marked below the error line. Values represented mean ± SE. *BW* body weight, *SR* survival rate, *ADG* average daily gain, *FCR* feed conversion rate, *FEI* fattening efficiency index. Compared with the average value of the same year, *indicated *P* < 0.05; **indicated *P* < 0.01; ***indicated *P* < 0.001; ****indicated *P* < 0.0001.

It can be seen from Fig. 2 that the monthly data of the number of piglets sources, month of piglets purchasing, the feeding days and the ADG in 2020 and 2021 showed similar trends, while other factors did not. The BW of deaths declined in the second quarter (Fig. 2I). The BW of marketing pigs increased from January to May while decreasing in the following months (Fig. 2K). The ADG showed a significant increase in May (Fig. 2M). The FCR in the first half of the year was higher than that in the second half (Fig. 2N). In 2020, the FEI was the highest in May and the lowest in December, while in 2021, the FEI was the highest in January (Fig. 2O).

Table 4 showed that there were significant differences between single and multiple sources, except for the feeding days in 2020 and 2021, the month of piglets purchasing in 2021, and the age of piglets in 2020. Specifically, piglets sources, number of piglets, feed consumption, number of deaths, number of marketing pigs and FCR were higher in multiple piglets sources. Instead, BW of piglets, age of piglets, BW of deaths, SR, BW of marketing pigs and ADG were lower, which directly led to the FEI of multiple sources being significantly lower than that of a single source.

**Table 4:** Productive performance of 2142 single source batches versus 450 multiple sources batches of commercial pigs in 2020 and 2146 single source batches versus 1120 multiple sources batches in 2021.

	Month of piglets purchasing^a^				Number of piglets sources				Number of piglets				BW of piglets			
	2020		2021		2020		2021		2020		2021		2020		2021	
	Single	Multiple	Single	Multiple	Single	Multiple	Single	Multiple	Single	Multiple	Single	Multiple	Single	Multiple	Single	Multiple
Purchased piglets																
Average	0.5	2.0	− 2.6	− 2.6	1.0	2.6	1.0	3.0	1,038.3	1,970.0	1,002.8	1,592.0	8.5	7.2	7.8	6.9
SD	6.7	5.7	6.6	6.6	0.0	1.1	0.0	1.4	703.9	1,497.0	814.0	1,154.1	2.2	2.1	1.9	1.5
Median	3.0	4.0	1.0	1.0	1.0	2.0	1.0	2.0	842.0	1,569.5	798.5	1,269.5	8.0	6.4	7.3	6.6
Min	− 5.0	− 5.0	− 5.0	− 5.0	1.0	2.0	1.0	2.0	120.0	191.0	100.0	123.0	4.4	4.0	4.2	4.1
Max	8.0	8.0	8.0	8.0	1.0	10.0	1.0	14.0	7,993.0	9,448.0	10,960.0	10,556.0	15.0	14.7	15.0	14.5
*P* value	< 0.0001****		0.8499		< 0.0001****		< 0.0001****		< 0.0001****		< 0.0001****		< 0.0001****		< 0.0001****	

	Age of piglets				Feed consumption				Number of deaths				BW of deaths^b^			
	2020		2021		2020		2021		2020		2021		2020		2021	
	Single	Multiple	Single	Multiple	Single	Multiple	Single	Multiple	Single	Multiple	Single	Multiple	Single	Multiple	Single	Multiple
Feeding																
Average	27.9	27.6	34.1	32.1	298,341.4	522,405.2	293,234.8	425,662.2	102.5	213.6	135.0	273.8	38.4	34.5	35.6	31.3
SD	9.1	8.5	9.6	8.9	199,754.1	397,138.6	233,693.8	302,949.4	125.7	245.8	153.9	269.2	17.1	14.0	18.1	15.4
Median	23.0	24.5	33.0	31.0	242,297.0	409,230.0	231,300.0	334,101.5	60.0	138.0	88.0	197.0	36.1	32.1	32.7	29.3
Min	18.0	18.0	18.0	18.0	28,420.0	30,500.0	25,660.0	36,300.0	0.0	0.0	0.0	4.0	5.4	6.3	2.1	3.8
Max	69.0	65.0	70.0	68.0	2,349,950.0	2,898,320.0	3,617,130.0	2,056,020.0	1240.0	2910.0	1434.0	2792.0	111.8	89.3	179.7	121.6
*P* value	0.5367		< 0.0001****		< 0.0001****		< 0.0001****		< 0.0001****		< 0.0001****		< 0.0001****		< 0.0001****	

	Month of marketing				SR				Number of marketing pigs				BW of marketing pigs			
	2020		2021		2020		2021		2020		2021		2020		2021	
	Single	Multiple	Single	Multiple	Single	Multiple	Single	Multiple	Single	Multiple	Single	Multiple	Single	Multiple	Single	Multiple
Marketing																
Average	8.3	9.3	6.6	6.9	90.8%	89.5%	87.2%	83.3%	935.8	1756.4	867.7	1319.0	121.3	114.1	121.6	116.6
SD	3.3	2.9	3.5	3.4	6.7%	6.8%	8.1%	8.5%	623.2	1329.7	699.9	950.9	14.8	14.9	16.5	15.6
Median	9.0	10.0	7.0	7.0	92.7%	91.2%	88.8%	84.6%	762.5	1374.5	673.0	1051.5	122.0	115.5	122.0	118.2
Min	1.0	1.0	1.0	1.0	65.3%	66.9%	65.0%	65.2%	111.0	134.0	100.0	110.0	65.3	65.7	65.8	69.6
Max	12.0	12.0	12.0	12.0	100.0%	100.0%	100.0%	100.0%	7672.0	8553.0	9589.0	7764.0	179.6	147.7	171.2	169.6
*P* value	< 0.0001****		0.0290*		0.0002***		< 0.0001****		< 0.0001****		< 0.0001****		< 0.0001****		< 0.0001****	

	Feeding days				ADG				FCR				FEI			
	2020		2021		2020		2021		2020		2021		2020		2021	
	Single	Multiple	Single	Multiple	Single	Multiple	Single	Multiple	Single	Multiple	Single	Multiple	Single	Multiple	Single	Multiple
Growth performance																
Average	164.4	165.2	178.0	179.2	0.68	0.64	0.63	0.60	2.79	2.73	2.94	2.89	227.9	215.7	194.2	179.0
SD	17.8	15.2	22.0	21.6	0.08	0.07	0.07	0.06	0.37	0.32	0.47	0.38	48.8	46.5	48.2	42.0
Median	164.0	165.5	178.0	180.0	0.69	0.65	0.64	0.61	2.73	2.69	2.86	2.82	232.1	223.3	195.4	179.8
Min	120.0	120.0	120.0	120.0	0.31	0.37	0.32	0.35	2.00	2.05	2.00	2.00	47.2	64.8	46.3	65.2
Max	263.0	207.0	271.0	262.0	0.90	0.83	0.89	0.79	4.99	4.84	5.00	5.00	374.2	333.7	359.5	360.8
* P* value	0.3862		0.1412		< 0.0001****		< 0.0001****		0.0007***		0.0009***		< 0.0001****		< 0.0001****	

*SR* survival rate, *ADG* average daily gain, *FCR* feed conversion rate, *FEI* fattening efficiency index.

^a^Month of piglets purchasing from last year was recorded as a negative number.

^b^When number of deaths was zero, BW of deaths was recorded as blank.

*indicates *P* < 0.05; **indicates *P* < 0.01; ***indicates *P* < 0.001'; ****indicates *P* < 0.0001.

## Discussion

A new productive evaluation indicator for commercial pigs, FEI, was proposed. FEI, as a single comprehensive indicator, can be used for daily/phase management of batches. Within the range of 5–150 kg commercial pigs, a high FEI meant a balance among a low death rate, good BW-gain speed and reasonable feed consumption. In this study, the FEI in May 2020 was the highest (Fig. 2O), the corresponding ADG was the highest (Fig. 2M), the FCR was the lowest (Fig. 2N), and the SR was above the annual average value (Fig. 2L). Similarly, in January 2021, the FEI was the highest (Fig. 2O), the corresponding ADG was the highest (Fig. 2M), the FCR was the lowest (Fig. 2N), and the SR was the highest (Fig. 2L). Therefore, it fully reflected the comprehensive fattening efficiency of commercial pigs, and avoided the bias of single indicators. When applied, the farm manager can learn useful experience from the top 5–10% FEI batches or summarize problems from the bottom 10–20% batches, and improvement measures are formulated and implemented to improve the following FEI of commercial pig batches.

Comparing the growth performance of the whole batches in 2021 and 2020, we found that on the premise of no significant difference in the feed consumption and the BW of marketing pigs, the feeding days increased by 13.9 days and the ADG decreased by 0.06 kg, resulting in a higher FCR, which indicated that the growth performance of commercial pigs in 2021 was worse than that in 2020 (Table 3). The results may be caused by the superposition of multiple factors and this should be confirmed by further research.

*First, it is prohibited to add antibiotics to the feed.* Feed not only accounted for the largest part of the cost, but also had a significant impact on the growth quality and health of pigs^8^. The addition of a prophylactic dose of antibiotics to the feed can improve the productive performance in a way that promoted growth, especially for weaned piglets, which was a simple way to avoid or reduce the risk of disease^9,10^. However, in consideration of public health risks, the EU implemented a ban on antibiotics as growth promoters in 2006^11^. On July 1, 2020, the Ministry of Agriculture and Rural Affairs of China required feed manufacturers to stop producing commercial feed containing growth-promoting additives (except traditional Chinese medicine). The feed in stock can be sold and used until December 31, 2020^12^. In this study, it was worth considering whether this policy affected the health of the pig herds in 2021 and led to an overall decline in productive performance. *Second, lighter weaning BW.* Although the overall purchase of piglets was older in 2021, the piglets were light in the BW (Table 3). The weaning BW was one of the basic characteristics that determined lifetime growth performance^13,14^. The BW at weaning and growth rate in the first week of nursing played an important role in subsequent performance^15^. Cabrera et al.^16^ found that at the age of 20 days weaning pigs weighing 5.0–5.9 kg reached 125 kg earlier than those weighing 4.1–5.0 kg. The pigs with a higher weaning BW reached the marketing BW 9–15 days earlier^17^. Our study found the average weaning age in 2021 was 5.6 days older than that in 2020 (Table 3). Although Faccin et al.^14^ pointed out that the increase in weaning age provided better productive performance and a higher adaptive level to weaning, in this study the average BW of piglets was lighter (8.3 kg versus 7.5 kg). It was doubtful that there may be problems with the health or growth performance of the pig herds. From the internal data management system, we found that the proportion of gilts in 2021 was 21.8% higher than that in 2020 (data not shown), which meant that the proportion of primiparous sows in 2021 was higher. As we all know, the reproductive performance of primiparous sows was lower than that of multiparous sows, which was reflected in a lower farrowing rate and a higher number of born alive piglets per litter^18,19^. Piglets farrowing by young sows were more likely to suffer from diseases, mainly because of the low availability of high-quality colostrum and milk, which meant lower passive immunity^20^. Consequently, a higher gilts proportion may affect the quality of piglets in 2021 to some extent. *Third, multiple sources or mixed feeding at different stages.* Influenced by ASF and the decision to combine farms, the number of sows in 2021 was only 54.05% of that in 2020, resulting in a sharp decrease in the number of weaned piglets on a breeding farm. The proportion of multiple sources increased from 17.36% in 2020 to 34.29% in 2021 (Table 4). Hence, the fattening farms were obliged to mix piglets from multiple sources or at different ages, which could contribute to increasing the risk of cross infection and bio-security, further affecting the growth performance^21,22^.

Poor productive performance of commercial pigs brought huge economic losses to large-scale breeding enterprises. The marginal benefit of each pig was mainly determined by the cost of piglets, feed and the value of the carcass. Under the condition of consistent BW of marketing pigs, lighter BW, more feeding days and a higher FCR meant a lower marginal benefit^23^. In addition to the worse growth performance, the number of deaths in 2021 was higher, and the average BW of deaths was lighter, which led to a decline in the SR. This may be due to the light BW of weaning piglets and more sources of piglets. The increase in the feeding days reduced the utilization rate of facilities and increased the fixed cost per pig^24^. Besides, deaths after weaning were also a wasteful investment in feed costs^25^.

The target pig farms of existing commercial pig-related studies were different from each other in terms of piglets source, herd size, farm design, environmental sanitation, herd health and feeding management^26^. The observed differences in farm performance were more likely to be the result of environmental health and farm management^25^. In our study, all pig batches came from branches throughout the country under the unified management of the group. They had similar construction standards, staff training standards, pig health management measures and bio-security prevention and control requirements, thus reducing the impact of external factors and more reliably and objectively reflecting the monthly/annual production performance and production efficiency of commercial pig batches on large-scale pig farms. Despite all this, the monthly data of different productive factors were still affected by the location, climate, batch scale (2592 versus 3266), pig herd health, the company's strategic decisions and market supply and demand, resulting in the monthly trend of fluctuation. As autumn and winter usher in Chinese intensive festivals, such as the Mid-Autumn Festival, the National Day, the New Year's Day, and the Spring Festival, these holidays promote the peak consumption of pork and provoke market demand. After the average BW of marketing pigs reaches the marketing standard, sales will be arranged as soon as possible, so that the feeding days are relatively short. Table 1 presented that the FCR of commercial pigs was higher in the later fattening stage, while Fig. 2F revealed that the average feeding days from March to June were the longest in the year, exceeding 185 days, which may be the main reason for the high FCR in the first half of the year. As far as we know, there is almost no evaluation study on the monthly production performance of commercial pigs at present.

In order to reduce the wasteful productive costs and economic benefits of pig farms, managers must maximize the fattening efficiency of commercial pigs^27^. In our study, we found five productive factors with the highest correlation with FEI (Table 2). Targeted improvement of these factors may help improve the productivity of commercial pigs, including appropriate nutrition, reasonably supplemented additives, good care at the early nursing stage, special attention to slow-growing pigs, pig health management (control and prevention of viral infections and secondary bacterial infections), correct housing conditions (environmental temperature and ventilation), available bio-security measures, appropriate immunization procedures and regular staff training^14,20,28–34^.

This study ignored the impact of disease and climate, because the internal data system did not record the related information on commercial pigs. Some studies have shown that the disease will adversely affect the productive performance of commercial pigs. For example, porcine epidemic diarrhea virus (PEDV) infection was the main cause of death in piglets^35^. Exposure to porcine reproductive and respiratory syndrome virus (PRRSV) and influenza type A virus of swine (IAV-S) led to lethargy, reduced growth rate, increased mortality and morbidity^36^. Especially in the case of multiple infections, the symptoms worsened. In terms of climate, Ranaldo et al.^37^ showed that the growth performance of 90 kg fattening pigs in tropical areas varied with the seasons. Nevertheless, few data points can confirm this or quantify its impact.

## Conclusions

Our study revealed that FEI may be suitable for evaluating the fattening efficiency of commercial pigs in batches. Factors related to FEI included ADG, FCR, SR, number of deaths, feeding days and BW of marketing pigs. In general, the annual and monthly productive performance in 2021 was worse than that in 2020. There was rarely a similar trend in monthly productive data for two consecutive years. The multiple piglets’ sources had a bad effect on the productive performance.

## Data Availability

The datasets generated and/or analysed during the current study are not publicly available due all data comes from the company’s encrypted internal data management system and contains sensitive content, but are available from the corresponding author on reasonable request.
